# Acetone Vapor-Sensing Properties of Chitosan-Polyethylene Glycol Using Surface Plasmon Resonance Technique

**DOI:** 10.3390/polym12112586

**Published:** 2020-11-04

**Authors:** Fahad Usman, John Ojur Dennis, E. M. Mkawi, Yas Al-Hadeethi, Fabrice Meriaudeau, Yap Wing Fen, Amir Reza Sadrolhosseini, Thomas L. Ferrell, Ahmed Alsadig, Abdelmoneim Sulieman

**Affiliations:** 1 Department of Fundamental and Applied Sciences, Universiti Teknologi PETRONAS, Seri Iskandar 32610, Perak, Malaysia; 2 Department of Physics, Faculty of Science, King Abdulaziz University, Jeddah 21589, Saudi Arabia; emrzog@kau.edu.sa (E.M.M.); yalhadeethi@kau.edu.sa (Y.A.-H.); 3 ImViA EA 7535, Team IFTIM, Université de Bourgogne, 21000 Dijon, France; 4 Department of Physics, Universiti Putra Malaysia, Serdang 43400, Selangor, Malaysia; yapwingfen@upm.edu.my; 5 Institute of Advanced Technology, Universiti Putra Malaysia, Serdang 43400, Selangor, Malaysia; amir1348@gmail.com; 6 Department of Physics and Astronomy, University of Tennessee, 401 Nielsen Physics Building and Joint Institute for Materials Research 1408 Circle Drive Room 219 2641 Osprey Way, Knoxville, TN 37996, USA; tferrell@utk.edu; 7 Department of Physics, Universita di Trieste, Piazzale Europa, 1, 34127 Trieste, Italy; modyalsadig@gmail.com; 8 Radiology and Medical Imaging Department, College of Applied Medical Sciences Prince Sattam bin Abdulaziz University, P.O. Box 422, Alkharj 11942, Saudi Arabia; a.sulieman@psau.edu.sa

**Keywords:** surface plasmon resonance sensor, acetone vapor detection, diabetes, chitosan-polyethylene glycol film, non-invasive

## Abstract

To non-invasively monitor and screen for diabetes in patients, there is need to detect low concentration of acetone vapor in the range from 1.8 ppm to 5 ppm, which is the concentration range of acetone vapor in diabetic patients. This work presents an investigation for the utilization of chitosan-polyethylene glycol (PEG)-based surface plasmon resonance (SPR) sensor in the detection of trace concentration acetone vapor in the range of breath acetone in diabetic subjects. The structure, morphology, and elemental composition of the chitosan-PEG sensing layer were characterized using FTIR, UV-VIS, FESEM, EDX, AFM, and XPS methods. Response testing was conducted using low concentration of acetone vapor in the range of 0.5 ppm to 5 ppm using SPR technique. All the measurements were conducted at room temperature and 50 mL/min gas flow rate. The sensor showed good sensitivity, linearity, repeatability, reversibility, stability, and high affinity toward acetone vapor. The sensor also showed better selectivity to acetone compared to methanol, ethanol, and propanol vapors. More importantly, the lowest detection limit (LOD) of about 0.96 ppb confirmed the applicability of the sensor for the non-invasive monitoring and screening of diabetes.

## 1. Introduction

Diabetes has been ranked among the top deadliest diseases globally [1,2]. In 2019, the people suffering from diabetes were estimated to be 9.3% (463 million people) globally. Unfortunately, the number has been projected to rise to around 700 million by 2045 [2,3,4]. World Health Organization (WHO) has described diabetes as the major culprit for blindness, kidney failure, heart attacks, stroke, and lower limb amputation [3]. Currently, diabetes is being diagnosed through blood glucose monitoring. [2]. However, the requirement of drawing blood samples makes the method invasive, painful, inconvenient, and an avenue of infectious diseases as well as damaging tissues [5,6]. This paves a way to investigating more convenient ways.

Fortunately, a high positive correlation has been reported between exhaled breath acetone and blood glucose [7]. This makes the relative level of exhaled acetone a suitable biomarker in detecting diabetes with a method of non-invasive monitoring [8]. Healthy subjects are reported to possess very low (0.1–0.8 ppm) acetone concentration while it might be elevated (1.8–5.0 ppm) in patients suffering from diabetes [7].

Numerous devices have been employed for the detection of acetone vapor at low concentrations. This includes conventional devices such as gas chromatography-mass spectrometry (GC-MS) and selective ion flow tube mass spectrometry (SIFT-MS). Despite the good sensitivity of these devices, the development and acceptance is hindered by their sophisticated instrumentation, nonreal-time measurement, expense, requirement of trained personnel, and scarcity [5]. On the other hand, metal oxide semiconductor (MOS)-based sensors have been reported to be the most investigated sensors in terms of low-concentration acetone vapor sensing [8]. However, these sensors are mostly operated at high temperature in addition to the selectivity and stability issues [8,9].

Optical sensors were reported to be more promising due to their greater sensitivity, electrical passiveness, freedom from electromagnetic interference, wide dynamic range, nonrequirement of reference electrode, freedom from electrical hazards, relatively high stability, the multiplexing capabilities, and the potential for higher-information content relative to that of electrical transducers [10]. In addition, another group of optical biosensors, surface plasmon resonance (SPR) sensors, has attracted many investigations due to their high sensitivity, real-time measurement, label-free measurement, and nonrequirement of electrodes [11,12,13]. These SPR sensors feature ultra-sensitive and selective detection when the surface is functionalized with certain materials depending on the analyte [11,12,13,14,15].

Studies have confirmed the promising applicability of chitosan-based materials in the detection of acetone vapor down to parts per million (ppm) levels [16]. Chitosan is an abundant material obtained from the de-acetylation of chitin. It is hydrophilic, biocompatible, moldable, biodegradable, renewable, nontoxic, cheap, and is a good absorbent material [17]. In addition, its amine and hydroxyl functional could play a vital role during interaction between an analyte and the surface of a chitosan-based material [18,19]. Moreover, addition of another polymer, polyethylene glycol (PEG) to chitosan, has been reported to increase the mechanical strength and biocompatibility of chitosan film [16,20].

This work was aimed at investigating acetone vapor-sensing properties of chitosan-PEG blend for possible non-invasive monitoring and screening of diabetes using the SPR technique. The performance measurements such as sensitivity, repeatability, binding affinity, and selectivity of the proposed sensor were explored and the results are reported here. Based on our literature survey, reports on the investigation of low concentrations of acetone vapor using SPR techniques are lacking.

## 2. Materials and Methods

### 2.1. Chemicals

Chitosan, polyethylene glycol (PEG), acetic acid, acetone (99%), ethanol, methanol, and propanol were all supplied by Avantis chemicals supply, Ipoh-Perak, Malaysia from Merck (Darmstadt, Germany) and Sigma Aldrich (St. Louis, MO, USA). All the chemicals were analytical grade.

### 2.2. Fabrication of Au/Chitosan-PEG SPR Sensor Film

Gold (Au) film was deposited on cleaned microscopic glass slips, Menzel-Glaser, Braunscheig, Germany, using a sputter coater, SC7640. The sputter coater was set at 20 mA and 67 s in order to achieve the optimal gold thickness of about 50 nm [15]. The solution of chitosan-PEG blend was prepared by dissolving 500 mg of the chitosan powder in 9 mL of 2% acetic acid glacial. Then, 10 mg of PEG was dissolved in 1 mL deionized water. The two separate solutions were later mixed. In addition, the mixture was stirred for 24 h in order to obtain a homogeneous solution of the blend. The thin layer of the chitosan-PEG was deposited on top of the gold-coated substrate using POLOS™ spin coater at 6000 rpm and 30 s. The thin film of the Au/chitosan-PEG was then kept in oven at 40 °C.

### 2.3. Characterization

FTIR characterization was conducted using FTIR spectrometer (Bruker Instruments, model Aquinox 55, Ettlingen, Germany) in the 4000–400 cm^−1^ range. The UV-VIS absorption and transmission spectra were obtained using Cary 100 UV-Vis Spectrophotometer from Agilent Technologies (Santa Clara, CA, USA). The surface morphology was studied using field emission scanning electron microscopy (FESEM) with images recorded by variable pressure field emission scanning electron microscope (VPFESEM), Zeiss Supra55 VP (Oberkochen, Germany). In addition, the energy-dispersive X-ray (EDX or EDS) was also conducted in order to determine the constituent elements for the blends. The thickness measurement was conducted using a surface roughness tester (SV-mutitoyo-3000, Mutitoyo, Aurora, IL, USA) and a surface profiler, AMBIOS, XP-200 based (AMBIOS, Santa Cruz, CA, USA) on a scratch following deposition of the film. An AFM study was conducted in order to investigate the surface roughness and coverage of the films. The functional groups of constituent materials present on the surface of the sensing layers were investigated by X-ray photoelectron spectroscopy (XPS, Thermo logical, K-alpha, Waltham, MA USA) in order to evaluate the interaction mechanism between the sensing layer and the acetone vapor.

### 2.4. SPR Measurement

**Figure 1:**
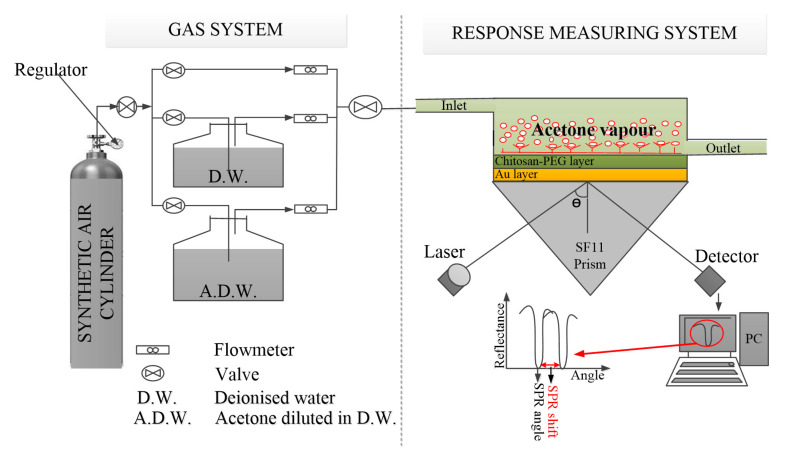
Sketch diagram of the entire surface plasmon resonance (SPR) response testing setup.

The experimental characterization was conducted using the setup illustrated in Figure 1. The details of the setup are contained in the picture illustrated in Figure S1. The setup is based on Kretschmann configuration. Typically, an Au/chitosan-PEG sensor film was attached onto the base of the SF11 prism using a Norland index matching liquid. The prism with attached sensor film was then placed on an optical stage for control and in order to allow the light to reach the gold film from interior through one face of the prism. At a specific angle of incidence, the SPR angle, the intensity of the light wave reflected from the other face was reasonably reduced. This is the SPR response, which was recorded by a silicon photodiode detector. The signal was then processed by a lock-in amplifier (SR530) and it was displayed as a sharp dip on a PC. The SPR angle is sensitive to 1 milliradian changes.

In addition, the gas was conveyed to a stainless-steel gas measuring cell attached to the sensing layer by a plastic tube. This conveyance was optimally controlled by the mass flow meters and valves, as shown in Figure 1 and Figure S1. The temperature and the relative humidity were monitored by humidity/temperature meter, HT-601C. All the experiments were conducted at room temperature. The optimum flow rate was explored in the range of 50–250 mL/min.

## 3. Results

### 3.1. Structural, Morphological, and Chemical Compositional Characterization of the Sensing Layer

**Figure 2:**
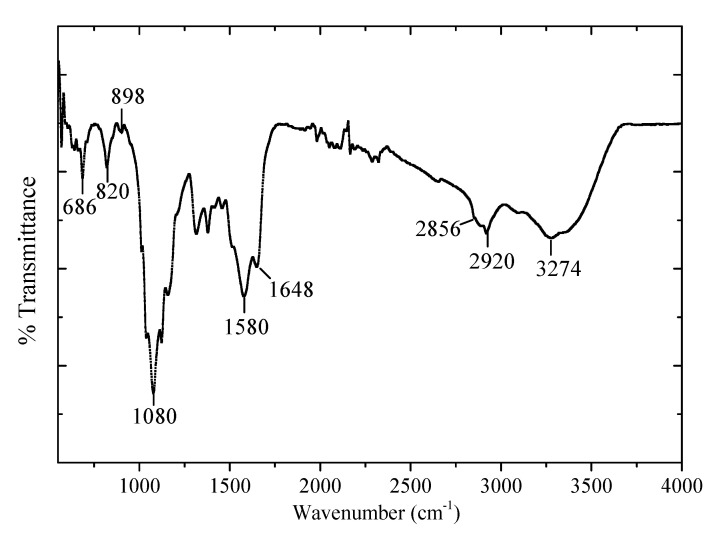
FTIR spectra of chitosan-Polyethylene glycol blend.

Figure 2 depicts the FTIR spectrum of the chitosan-PEG blend. The broad peak at 3274 cm^−1^ is due to the N–H stretching and O–H stretching vibrations of the chitosan and PEG [21]. The broadness is further confirming the association of the two polymeric materials. Moreover, the peaks at 2920 and 2856 cm^−1^ are attributed to the asymmetric and symmetric C–H stretching, respectively [22,23]. The peak at 1080 cm^−1^ could be attributed to the C–O stretching of ether group for PEG while the peaks 898 and 820 cm^−1^ could also be attributed to the similar PEG characteristics’ peaks observed previously [21,22].

**Figure 3:**
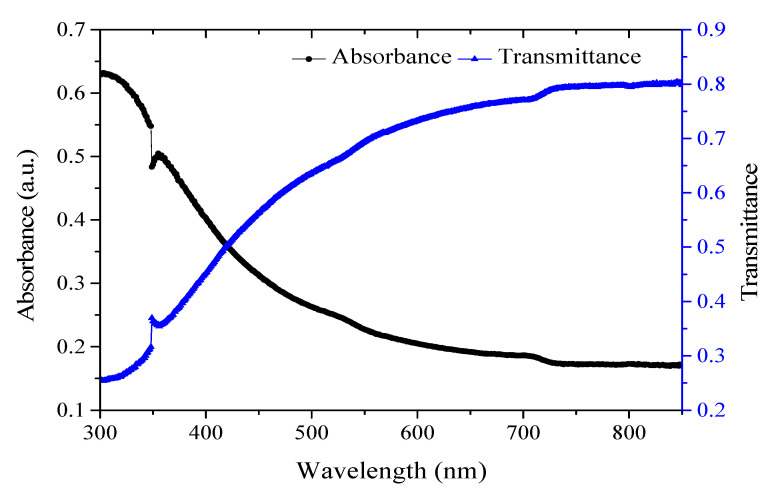
UV-VIS absorption and transmittance spectra of chitosan-PEG blend.

Figure 3 shows the absorption and transmittance spectra of the chitosan-PEG. It is reported that chitosan featured no absorption peak within 300–900 nm [24]. However, the minor peak observed around 350–400 nm could be attributed to the presence of PEG [25,26]. On the other hand, the transmittance value of the material indicates its promising application in the visible range [27].

**Figure 4:**
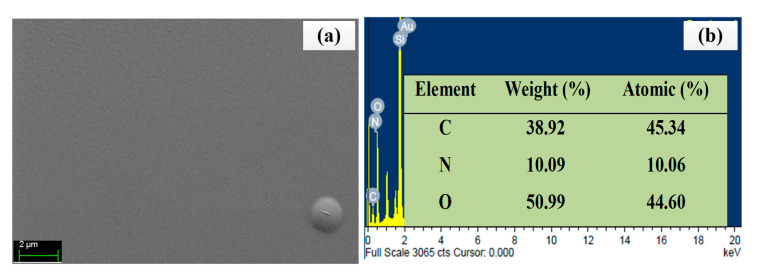
(**a**) FESEM image of chitosan-PEG, (**b**) energy-dispersive X-ray (EDX) spectrum and elemental composition of chitosan-PEG.

The surface morphology and EDX spectrum of the chitosan-PEG are shown in Figure 4a,b, respectively. Figure 4a shows no obvious feature for the chitosan-PEG surface. This could be due to the flatness nature of chitosan films and it is consistent with the previous work [28,29]. In addition, it confirms the absence of bubbles in the chitosan-PEG blend [29].

As shown in Figure 4b, the higher oxygen (%) contents in the chitosan-PEG confirm the abundance in OH functional group, which has the potential to increase the analyte-sensing layer interaction [30]. In addition, EDX could penetrate down to about 2000 nm [31]. As such, Si and Au could be observed, which originated from the substrates and the gold film, respectively.

The surface roughness of the glass substrate, gold layer, and the chitosan-PEG-coated gold layer were derived from the AFM surface morphological images shown in Figure S2a–c, respectively. The surface features are in consistence with the respective FESEM image. Based on the roughness data in Table 1, both the roughness average (Ra) and Root Mean Square (RMS) roughness values for the glass substrate and gold layer could lead to a good SPR sensor [32]. The Ra roughness value for the chitosan-PEG films is 5.87 nm while its RMS value is 9.29 nm. This higher roughness value could improve the response of the sensor due to the potentially increased adsorption capability of rough surfaces [33].

The optimum gold thickness for the SPR generation is around 50 nm [15]. As shown in Figure S3a,b, similar values, 47.458 nm and about 50 nm, were obtained for the surface profiler- and surface roughness tester-based measurements, respectively.

### 3.2. Acetone Detection Measurement

#### 3.2.1. Optimization of Experimental Conditions

Prior to the response testing, the temperature and the relative humidity of the optimal flow rate were monitored using a humidity/temperature meter, HT-601C. Subsequently, these conditions were maintained with the aid of a protective fabric, illustrated in Figure S1.

**Figure 5:**
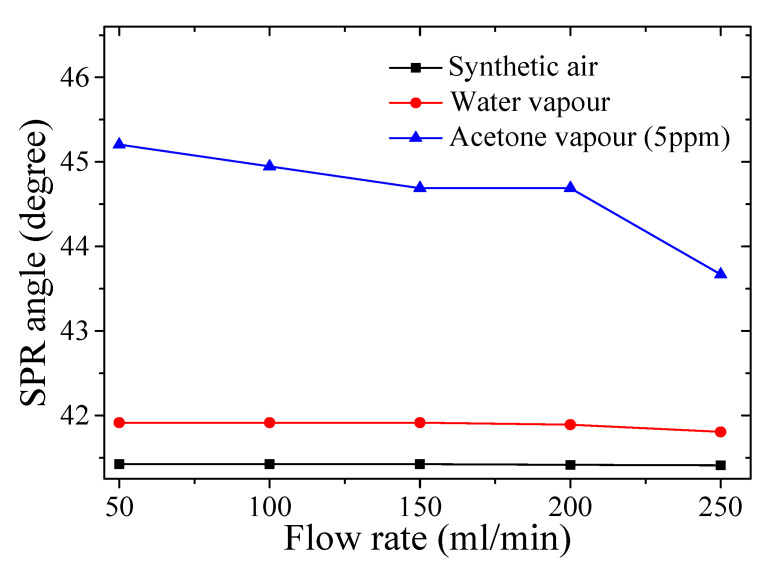
SPR angle vs. flow rate on the response of chitosan-PEG-based SPR sensor in synthetic air (carrier gas), water vapor, and 5 ppm acetone vapor.

Figure 5 shows measured SPR angle at various flow rates in the range of 50–250 mL/min for the synthetic air, water vapor, and 5 ppm acetone vapor. It was observed that the highest SPR angle was recorded at the flow rate of 50 mL/min for all the analytes at the recorded chamber temperature of about 29.0 °C. The measured relative humidity (RH) % values in the chamber for the synthetic air (carrier gas), water vapor, and 5 ppm acetone vapor were about 20.09% RH, 92.81% RH, and 87.50% RH, respectively. As such, all the subsequent chitosan-PEG-based SPR measurements were conducted under these conditions.

#### 3.2.2. SPR Response on the Chitosan-PEG-Based Sensor to Different Acetone Vapor Concentrations in Air

**Figure 6:**
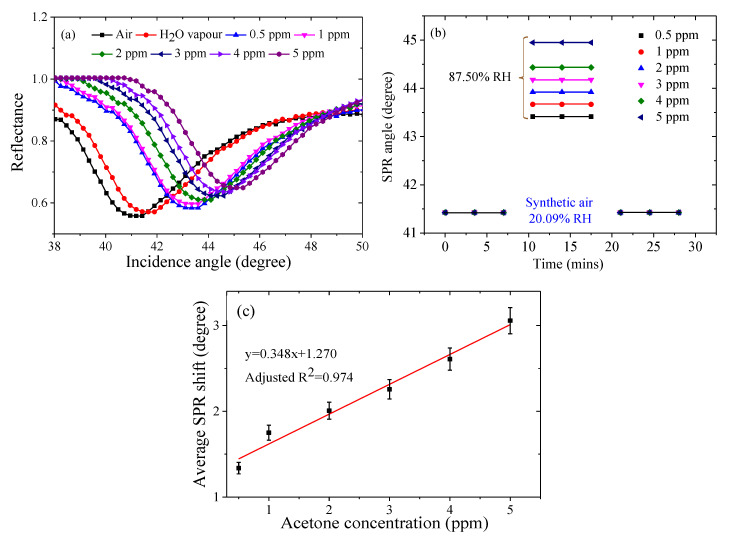
(**a**) Reflectance versus incident angle for the chitosan-PEG-based SPR response in dry air, water vapor, and the various concentrations of the acetone vapor from 0.5 ppm to 5 ppm, (**b**) SPR angle versus time of the single layer chitosan-PEG-based SPR sensor at different acetone vapor concentrations from 0.5 ppm to 5 ppm as compared to synthetic air levels, and (**c**) SPR angle shift versus acetone concentration from 0.5 ppm to 5 ppm.

Prior to the investigation of the chitosan-PEG-based sensor response to the various concentrations of acetone vapor, the steady condition for the SPR measurement and the restoration of the sensing layer were achieved by allowing 5-min exposure to the analytes and synthetic air, respectively [15]. The SPR response of chitosan-PEG sensing layer to dry air (synthetic air at 20.09% RH) different, water vapor (humidified air at 92.81% RH), and to acetone vapor concentrations from 0.5 ppm to 5 ppm in humidified air (at 87.50% RH) was measured, as shown in Figure 6a. A positive SPR shift was observed with the increase in the concentration of the acetone vapor. The SPR shift was due to changes in the surface plasmon properties of the gold film plus absorbate relative to the gold film alone, as caused by the optical properties of the absorbed analyte as well as the change of the refractive index of the sensing layer [15].

Figure 6b,c shows the graph of the SPR angle against time and calibration curve, respectively. The excellent repeatability and linearity infer the suitability of the device for acetone vapor sensing in the exhaled breath for diabetes monitoring and screening within the range of 1.8 ppm to 5 ppm in diabetic subjects [7]. The repeatability of the chitosan-PEG-based SPR sensor was assessed by the values of standard deviation and the coefficient of variation (COV) [15,34]. Based on the results presented in Table S1, the average standard deviation for the three replicas was about 0.054. In addition, the relative standard deviation (RSD) or the coefficient of variation (COV) value was found to be 0.123%. These indicate the repeatability of the measurement [35,36]. This behavior is further illustrated in Figure 6b.

In order to compute the calibration curve of the acetone detection of the chitosan-PEG-based SPR sensor, the effect of water vapor was eliminated, as shown in the last column of Table S1. The calibration curve is illustrated in Figure 6b, which shows a good linear response of the sensor. The linear regression analysis is governed by the Equation (1),

Δ*θ* = *kC* + *I* (1)

where Δ*θ* is the average SPR angle shift, *k* is the slope, which is the sensitivity in degree/ppm, *C* is the acetone concentration in ppm, and *I* is the intercept. Figure 6b indicates that the average SPR shift is linearly correlated to the acetone vapor concentration in air with a high correlation factor of 0.974 and with a corresponding sensitivity value of 0.348 degree/ppm.

#### 3.2.3. Thickness Variation of Layers and Lowest Detection Limit (LOD) of the Chitosan-PEG Films

**Figure 7:**
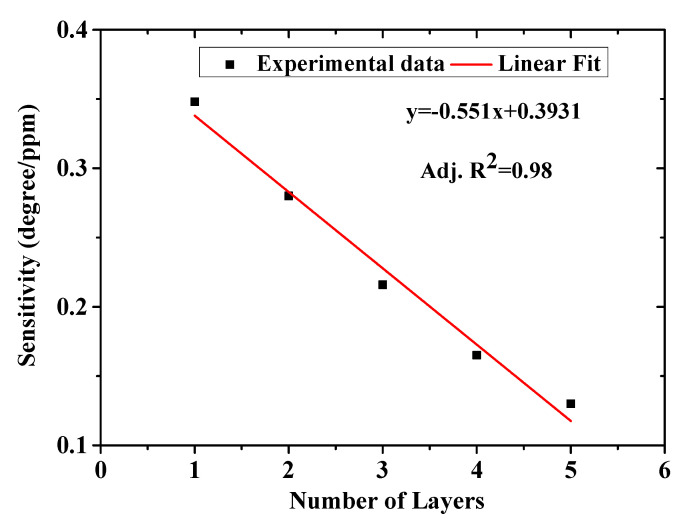
Sensitivity versus number of chitosan-PEG layers.

The result presented in Figure 6 is from a single layer of chitosan-PEG deposited at 6000 rpm for 30 s. In order to investigate the effect of layer thickness on the sensitivity of the chitosan-PEG-based SPR sensor, four different chitosan-PEG sensing layers with 2, 3, 4, and 5 layers deposited on top of the first layer to increase thickness were also prepared and tested. Figure S4a,b shows the effect of the number of layers on the SPR curves and the sensitivity, respectively. The results are summarized in Figure 7 and Table 2. It could be observed from Table 2 that the full width at half maximum (FWHM) increases with number of layers, which is attributed to the increase in the thickness of the chitosan-PEG sensing layer. From Figure 7 it can be observed that the sensitivity decreases with number of chitosan-PEG layers, which is in accordance with a result on the SPR detection of ethanol and isopropanol [37]. This could be due to the decrease of the penetration depth of the surface plasmon wave [12,14]. Based on the result presented in Figure 7 and Table 2, it could be concluded that single-layer chitosan-PEG-based SPR sensor is the best in terms of sensitivity, FWHM, and figure of merit (FOM).

The lowest detection limit (LOD) of the chitosan-PEG-based SPR acetone vapor sensor was estimated using the ratio 3σ/sensitivity [38], where *σ* stands for the standard deviation of the blank sample. The SPR curves of the blank sample and its values for one-layer chitosan-PEG-based SPR acetone vapor sensor are shown in Figure S5 and Table S2, respectively. The standard deviation (*σ*) of 10 replicas was evaluated to be about 0.0001. This gives the LOD value of about 0.96 parts per billion (ppb).

#### 3.2.4. SPR Angle Versus Time Graph of Single-Layer Chitosan-PEG-Based SPR Sensor for the Detection of Acetone Vapor

**Figure 8:**
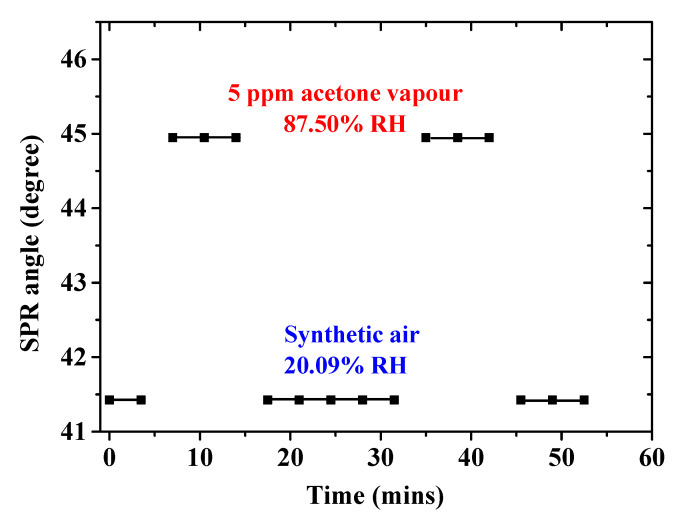
SPR angle versus time of the single-layer chitosan-PEG-based SPR sensor at 5 ppm acetone vapor concentration as compared to synthetic air levels.

The SPR angle of the single-layer chitosan-PEG-based SPR sensor was evaluated in order to investigate the recovery, response, stability, and reversibility of the measurements [39,40,41]. The graph is shown in Figure 8 as a plot of SPR angle as a function of time [15,42]. Unfortunately, our SPR system setup could not provide SPR angle measurement data faster than 3.5 min after each run due to the need to control and adjust some of its components manually. Therefore, accurate response and recovery time values could not be determined. However, it could be observed from Figure 8 that both the response and recovery times would be less than 1 min each after exclusion of the 3.5 min. Excellent reversibility and recovery of the sensor was observed when the supply of the 5 ppm acetone vapor was ceased and replaced by the introduction of the synthetic air. Other concentrations of the acetone vapor also showed similar characteristics (Figure 6b).

#### 3.2.5. Binding Affinity of Acetone toward SPR Sensor with Single Layer of Chitosan-PEG

**Figure 9:**
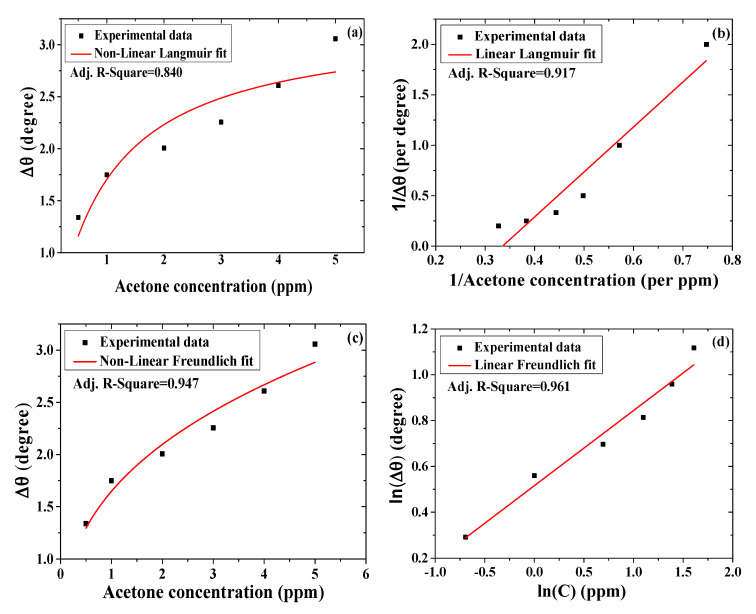
Binding affinity between the single-layer chitosan-PEG-based SPR sensor and various acetone concentrations fitted to (**a**) nonlinear Langmuir, (**b**) linear Langmuir, (**c**) nonlinear Freundlich, and (**d**) linear Freundlich isotherms models.

The investigation of the binding strength between the single-layer chitosan-PEG SPR sensor and acetone vapor was deduced from the plotting graph of the average SPR angle shifts as a function of the average acetone concentrations, shown in Figure 6c. This graph was fitted to the nonlinear and linear formats of the Langmuir and the Freundlich isotherm models, as shown in Equation (S1), (S2), (S3), and (S4), respectively [15,43]. The fits with low error value and higher correlation factor were regarded as the best [44]. Figure 9a–d shows the graphs for the nonlinear Langmuir fittings, linear Langmuir fittings, nonlinear Freundlich fittings, and linear Freundlich fittings, respectively. These results are presented in Table 3. As shown in Figure 9 and Table 3, the parameter Δ*θ* is the SPR shift, Δ*θ_max_* is the maximum SPR shift at saturation, *C* is the concentration of the analyte, and *K_D_* is the equilibrium dissociation constant. Affinity constant (*K_A_*) is the reciprocal of *K_D_*. In addition, 1/*n* is the heterogeneity factor [43,45]. A variation in the slope (1/*n*) between 0 and 1 is associated with a chemisorption process. When a slope above 1 is observed, then a physical absorption process is expected [46,47]. *K_F_* can be related to the strength of the adsorptive bond or adsorption capacity. Furthermore, Δ*θ*_max_ is measured in degree, *K*_A_ is measured in ppm^−1^, and *K*_D_ and *K*_F_ are measured in ppm [15,48].

The correlation factor values for the nonlinear Langmuir, linear Langmuir, nonlinear Freundlich, and linear Freundlich fittings are 0.84, 0.92, 0.95, and 0.96, respectively. This shows that the Freundlich model also fit better for the chitosan-PEG SPR sensing layer. Freundlich model also showed smaller values of standard error, reduced chi-square, and residual sum of squares. However, the error and variability values observed in the Langmuir are not reasonably high. As such, both the Langmuir and the Freundlich models could be used to describe the adsorption process on the surface of the chitosan-PEG sensing layer. In addition, the Δ*θ*_max_ value obtained was closer to the maximum shift of 3.057 ppm, depicted in Table S1 and Figure 6. In this regard, the linear Langmuir model showed the closest value (2.994 ppm) due to the lower standard error of its intercept (0.306) compared to nonlinear model (0.328). Furthermore, the *K*_A_ value, 1.12 ppm^−1^ (2.704 × 10^7^ M^−1^ or 1.120 × 10^3^ g/mg), for the nonlinear Langmuir model is more reliable for its low standard error compared to the linear model. Additionally, the higher *K*_A_ value compared to *K*_D_ value of 0.893 ppm indicates the greater affinity of the acetone toward the chitosan-PEG sensing layer [15].

For the Freundlich fittings, the linear format showed the best correlation, less variability, and lower error values, as shown in Table 3. As such, its result was considered against the nonlinear format, where it was observed that *K*_F_ and *n* values were 1.430 ppm and 3.049, respectively. The *K*_F_ value was equivalent to 5.921 × 10^−8^ M [48,49]. In addition, chemical adsorption process was expected to be dominant on the surface of the chitosan-PEG, since the slope (1/*n*) < 1 [46].

#### 3.2.6. Detection Mechanism and Selectivity Test of the Single-Layer Chitosan-PEG-Based SPR Acetone Vapor Sensor

**Figure 10:**
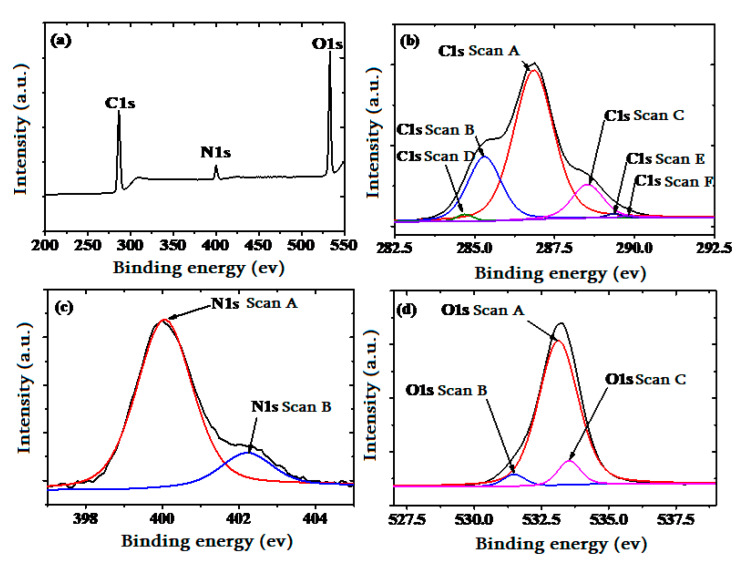
XPS spectra of the (**a**) survey scan, (**b**) C1s, (**c**) N1s, and (**d**) O1s, peaks for the single-layer chitosan-PEG blend thin film

The knowledge of functional groups on the surface of the chitosan-PEG sensing layer is required for the prediction of the dominant interaction mechanism and the reason for a selective detection of the acetone vapor [50], and this is also accomplished by XPS characterization. Its spectra are shown in Figure 10. The assignment of various peaks is summarized in Table S3. The presence of the carbon, nitrogen, and oxygen in XPS scan spectrum confirms the existence of the chitosan-PEG blend [51]. Furthermore, the C 1s scan of the chitosan-PEG blend was resolved to the binding energies (BEs) of 286.69 eV (C–OH), 284.90 eV (C–NH, C–NH_2_ or C=C), 288.35 eV (C=O), 285.31eV (Contamination, C–C or C–H), and about 289 eV (O–C=O) [52]. Furthermore, the O1s peak was resolved to three peaks, which include the BEs of 533.13 (C=O), 531.49 (C–OH), and 533.53 (C–O) [52]. The presence of hydroxyl (OH) and C–NH further confirms the presence of chitosan [51,53]. The N1s peak was resolved to two different peak Bes’ positions at 400.05 eV and 402.21 eV, which could be attributed to Pyrollic–N (–NH–) and Pyridinic–N (=N–), respectively [51]. These abundant functional groups would play a vital role in the selective acetone vapor detection.

Based on the result of the surface characterization, the interaction between the chitosan-PEG layer and the acetone vapor could be due to multiple mechanisms. But the dominant interaction mechanism was predicted using the adsorption study to be based on chemisorption process. The chemisorption process is described by a two-step process. First, the exposure of the chitosan-PEG sensing layer to air led to the chemisorption of oxygen. This chemisorbed oxygen captured electron from the conduction band of the chitosan-PEG, which consequently produced ionic oxygen species, as shown in Equations (2)–(5). In the second step, when the chitosan-PEG sensing layer was exposed to acetone vapor, it reacted with the ionic oxygen species, which consequently led to increase in conductivity due to the release of the captured electron back to the conduction band, which in turn altered the refractive index value. This process is described in a simplified form by Equation (6) [54,55]. Conductivity describes how fast an electric charge can pass through a material or medium. A physical field that surrounds the electric charges is called electric field. On the other hand, the ability to allow the passage of an electric field through a material can be described by the parameter of real part dielectric constant [56,57]. The complex refractive index (*n*) of a medium can be related to its complex dielectric constant (*ε_r_*) using the solution of Maxwell’s equation, shown in Equation (7) [58]. This indicates that *n* value increases with increase in *ε_r_* value. Based on this, it can be concluded that the movement of the captured electron back to the conduction band will increase the dielectric constant value, which will in turn increase the refractive index value.

O_2_(gas) ↔ O_2_(adsorbed) (2)

O_2_(adsorbed) + e^−^ ↔ O_2_^−^(adsorbed) (3)

O_2_^−^(adsorbed) + e^−^ ↔ 2O^−^(adsorbed) (4)

O^−^(adsorbed) + e^−^ ↔ O^2−^(adsorbed) (5)

CH_3_COCH_3_ (gas) + O^2−^ → CO_2_ (gas) + H_2_O (gas) + 2e^−^ (6)

(7) $$n=\sqrt{\varepsilon_{r}}$$

In addition, the hydrogen bond formation between the hydrogen of the NH group in chitosan-PEG and the oxygen from the CO group of the acetone could act as an electrical bridge for the electron transfer [29]. This would enhance the response of the SPR sensor by producing greater change in refractive index value.

#### 3.2.7. Selectivity of Chitosan-PEG-Based SPR Sensor to Acetone Vapor

**Figure 11:**
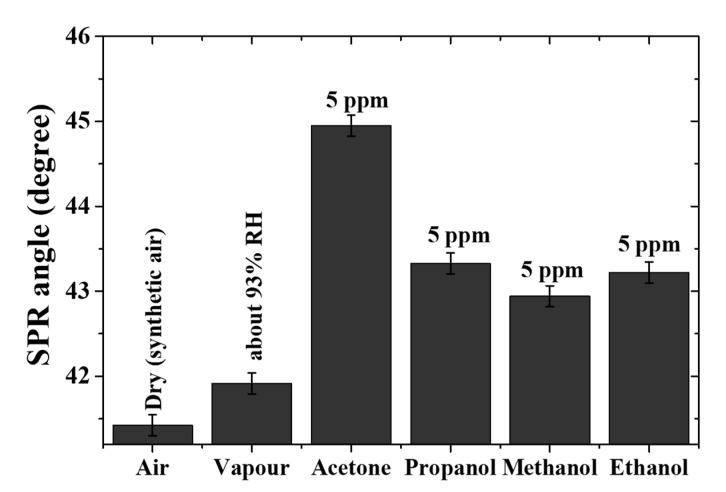
Selectivity of the chitosan-PEG sensing layer to 5 ppm acetone vapor compared to humidity, 5 ppm propanol, 5 ppm methanol, and 5 ppm ethanol vapors.

The cross-sensitivity (selectivity) of the single-layer chitosan-PEG SPR sensor to acetone was confirmed by investigating and comparing the response of the sensor to water vapor, 5 ppm propanol, 5 ppm methanol, and 5 ppm ethanol with that of 5 ppm acetone vapor. The selectivity graph is shown in Figure 11. It was observed that the maximum SPR angle in air, vapor (about 93% RH), acetone vapor, propanol vapor, methanol vapor, and ethanol vapor were 41.41, 41.95, 44.95, 43.95, 42.94, and 43.22 degrees, respectively. The exclusion of the humidity effect made the response of 5 ppm acetone to be about 33%, 66%, and 57% higher than that of 5 ppm propanol, 5 ppm methanol, and 5 ppm ethanol, respectively. The higher response of the chitosan-PEG SPR sensor to acetone could be attributed to the higher number of carbon atoms and the rate of evaporation [42,59]. The number of carbon atoms and the rate of evaporation for all the analytes are presented in Table 4 [42]. It could be observed that both the acetone and the propanol shared the same number of carbons, but acetone showed higher response due to its higher rate of evaporation. Furthermore, comparison among the alcohols (propanol, ethanol, and methanol) indicates the domination of the number of carbon atoms.

## 4. Conclusions

The detection of acetone vapor at low concentration using chitosan-PEG-based SPR sensor was investigated. The intention was to explore the possibility of using the SPR sensor in the non-invasive monitoring and screening of diabetes. The surface characterization confirmed the presence of important functional groups such as OH and amine that could lead to a highly sensitive and selective detection of acetone. Furthermore, the results indicated that the sensor could detect the acetone vapor down to 0.96 ppb with sensitivity value of about 0.35 degree/ppm. The achieved LOD is far less than the diabetes threshold (1.8–5 ppm). This confirms the potentiality of the sensor. In addition, the adsorption studies based on the Langmuir and Freundlich isotherm models indicated good affinity of the sensing layer to acetone. Also, the heterogeneity factor (1/*n*) of <1 predicted the chemisorption process to be the dominant interaction mechanism. These are in addition to the good selectivity against the interfering analytes, linearity, repeatability, and stability. As such, the chitosan-PEG-based SPR sensor could realize a non-invasive sensor for monitoring and screening of diabetes using the acetone vapor from exhaled breath.

## Figures and Tables

**Table 1:** Roughness parameters for the glass, gold, and the chitosan-PEG film surfaces.

Material	Ra (nm)	RMS (nm)
Glass	0.16	0.22
Gold	2.13	4.05
Chitosan-PEG	5.87	9.29

**Table 2:** Performance characteristics of chitosan-PEG SPR acetone vapor sensor based on 1, 2, 3, 4, and 5 deposited layers of chitosan-PEG films.

Number of Layers	FWHM (Degree)	Sensitivity (Degree/ppm)	FOM (per ppm)
1	4.86	0.348	0.07
2	5.12	0.280	0.05
2	7.81	0.216	0.03
4	8.85	0.165	0.02
5	indefinite	0.130	0

**Table 3:** Binding parameters of acetone vapor toward single-layer chitosan-PEG-based SPR sensor extracted from nonlinear Langmuir, linear Langmuir, nonlinear Freundlich, and linear Freundlich fittings.

Model	Format	Parameter	Value	Standard Error
Langmuir	Non-linear	Δ*θmax*	3.227	0.328
		1/*K_A_*	0.893	0.320
		Adj. R2	0.840	-
		Reduced chi-square	0.060	-
		K_A_	1.120	-
		K_D_	0.893	-
Langmuir	Linear	Residual sum of squares	0.160	-
		Adj. R2	0.917	-
		Intercept	0.334	0.306
		Slope	4.458	0.595
		Δθmax	2.994	-
		*K_A_*	0.075	-
		*K_D_*	13.333	-
Freundlich	Non-linear	*K_F_*	1.647	0.082
		*n*	2.874	0.337
		Adj. R2	0.947	-
		Reduced chi-square	0.020	-
Freundlich	Linear	Residual sum of squares	0.014	-
		Adj. R2	0.961	-
		Intercept	0.516	0.031
		Slope	0.328	0.030
		*K_F_*	1.430	-
		*n*	3.049	-

**Table 4:** Properties of the analytes extracted from a previous work [42].

Hydrocarbon	Chemical Formula	Carbon Number	Evaporation Rate *n*-butyl acetate = 1.0
Acetone	C_3_H_6_O	3	14.4
Ethanol	C_2_H_6_O	2	1.7
Propanol	C_3_H_8_O	3	1.3
Methanol	CH_4_O	1	4.1

## Supplementary Materials

The following are available online at https://www.mdpi.com/2073-4360/12/11/2586/s1. Figure S1: Picture of the experimental SPR setup. Table S1: Data for the chitosan-PEG-based SPR sensor. Figure S2: AFM images of glass, gold, and chitosan-PEG surfaces. Figure S3: Thickness of gold thin film deposited at 20 mA, 67s estimated using a (a) surface profiler and (b) surface roughness tester. Figure S4: (a) SPR curves of different layers of the chitosan-PEG-based SPR sensor, (b) SPR angle shift versus the acetone concentration. Figure S5: Blank SPR response. Table S2: Blank sample response, Langmuir and Freundlich adsorption equations (Equations (S1)–(S4)). Table S3: Assignment of the C1s, O1s, N1s, and S2p peaks for the chitosan-PEG thin layer.
