# Supplementary material for: Acetone Vapor-Sensing Properties of Chitosan-Polyethylene Glycol Using Surface Plasmon Resonance Technique
Source: Polymers (Basel). 2020 Nov 4;12(11):2586. doi: 10.3390/polym12112586 (PMC7694228; doi:10.3390/polym12112586)
Supplement: Supplementary file 1 [file polymers-12-02586-s001.docx]

Article

Acetone Vapor-Sensing Properties of Chitosan-Polyethylene Glycol Using Surface Plasmon Resonance Technique (Supporting document)

Fahad Usman ^1^,*, John Ojur Dennis ^1,^*, E. M. Mkawi ^2^, Yas Al-Hadeethi ^2^, Fabrice Meriaudeau ^3,^*, Yap Wing Fen ^4,5^, Amir Reza Sadrolhosseini ^5^, Thomas L. Ferrell ^6^, Ahmed Alsadig ^7^ and Abdelmoneim Sulieman ^8^

^1^ Department of Fundamental and Applied Sciences, Universiti Teknologi PETRONAS, Malaysia, Seri Iskandar, 32610, Perak, Malaysia

^2^ Department of Physics, Faculty of Science, King Abdulaziz University, Jeddah 21589, Saudi Arabia; emrzog@kau.edu.sa (E.M.M.); yalhadeethi@kau.edu.sa (Y.A.-H.)

^3^ ImViA EA 7535, Team IFTIM, Université de Bourgogne, Dijon, 21000, Dijon, France

^4^ Department of Physics, Universiti Putra Malaysia, Serdang, 43400, Selangor, Malaysia; yapwingfen@upm.edu.my

^5^ Institute of Advanced Technology, Universiti Putra Malaysia, Serdang, 43400, Selangor, Malaysia; amir1348@gmail.com

^6^ Department of Physics and Astronomy, University of Tennessee, 401 Nielsen Physics Building and Joint Institute for Materials Research 1408 Circle Drive Room 219 2641 Osprey Way, Knoxville, TN 37996 Tennessee, USA; tferrell@utk.edu

^7^ Universita di Trieste, Piazzale Europa, 1, 34127 Trieste, Italy; modyalsadig@gmail.com

^8^ Radiology and Medical Imaging Department, College of Applied Medical Sciences Prince Sattam bin Abdulaziz University, P.O.Box 422, Alkharj 11942, Saudi Arabia; a.sulieman@psau.edu.sa

***** Correspondence: fahatu11@gmail.com (F.U.); johndennis@utp.edu.my (J.O.D.); Fabrice.Meriaudeau@u-bourgogne.fr (F.M.)

**Figure S1.** Picture of the experimental SPR setup*.*

**Table S1.** Result for the measurement of chitosan-PEG SPR angle shift due to air, water vapour and the various concentrations of the acetone vapour (0.5-5 ppm).

| **Concentration of acetone vapour (ppm)** | **SPR angle, θ (degree)**  **1st Run** | **SPR angle, θ (degree)**  **2nd Run** | **SPR angle, θ (degree)**  **3rd Run** | **Average** | **Average σ** | **Coefficient of variation**  **(σ/Average)** | **SPR shift due to acetone, Δθ (degree)** |
| --- | --- | --- | --- | --- | --- | --- | --- |
| Air | 41.4244 | 41.4244 | 41.4691 | 41.4393 | 0.02107 | 0.00051 |  |
| H2O vapour | 41.9151 | 41.9151 | 41.9151 | 41.9151 | 0 | 0 | 0 |
| 0.5 | 43.4145 | 43.4338 | 42.9103 | 43.2529 | 0.24236 | 0.00560 | 1.3378 |
| 1 | 43.6673 | 43.6673 | 43.6590 | 43.6645 | 0.00391 | 0.00009 | 1.7494 |
| 2 | 43.9215 | 43.9215 | 43.9215 | 43.9215 | 0 | 0 | 2.0064 |
| 3 | 44.1777 | 44.1777 | 44.1575 | 44.1710 | 0.00952 | 0.00022 | 2.2559 |
| 4 | 44.4333 | 44.6893 | 44.4478 | 44.5235 | 0.11741 | 0.00264 | 2.6084 |
| 5 | 44.9472 | 44.9472 | 45.0229 | 44.9724 | 0.03569 | 0.00079 | 3.0573 |
|  |  |  |  | 43.4825 | 0.05375 | COV=0.00123 |  |

**Figure S2.** AFM surface morphological images for the (a) glass substrate, (b) gold thin film and the (c) chitosan-PEG thin film, respectively.

(a)

(b)

**Figure S3.** Result for the thickness measurement of a gold thin film deposited at 20 mA, 67s using (a) surface profiler, AMBIOS, XP-200 (b) surface roughness tester.

**Figure S4.** (a) SPR curves of different layers of chitosan-PEG based SPR sensor in synthetic air (1-5 layers) and (b) SPR angle shift versus the acetone concentration (0.5-5 ppm) for 1,2,3,4 and 5 layers of chitosan-PEG based SPR sensor.

**Figure S5.** SPR response of blank sample for the estimation of LOD.

**Table S2.** Blank sample response to 1 layer chitosan-PEG SPR acetone vapour sensor.

| **Number of runs** | **SPR angle (degree)** |
| --- | --- |
| 1 | 41.9151 |
| 2 | 41.9154 |
| 3 | 41.9151 |
| 4 | 41.9154 |
| 5 | 41.9151 |
| 6 | 41.9151 |
| 7 | 41.9151 |
| 8 | 41.9151 |
| 9 | 41.9151 |
| 10 | 41.9151 |

Adsorption studies

| (S2) | (S2) |
| --- | --- |
| $\frac{1}{\Delta\theta}=\left[\frac{1}{\Delta\theta_{\max}K_{A}}\right]\frac{1}{C}+\frac{1}{\Delta\theta_{\max}}$ | (S3) |
| $\Delta\theta=K_{F}C^{\left(\frac{1}{n}\right)}$ | (S4) |
| $\ln(\Delta\theta)=\ln(K_{F})+\frac{1}{n}\ln(C)$ | (S5) |

**Table S3.** Assignment of the C1s, O1s, and N1s peaks for the single layer chitosan-PEG composite thin film.

| **Name** | **Peak BE (eV)** | **Assignment** |
| --- | --- | --- |
| O1s A | 533.13 | C=O |
| O1s B | 531.49 | C-OH |
| O1s C | 533.53 | C-O |
| C1s A | 286.85 | C-O |
| C1s B | 285.31 | Contamination, C-C or C-H |
| C1s C | 288.53 | C=O/O-C=O |
| C1s D | 284.69 | C-NH, C- NH2 or C=C |
| C1s E | 289.36 | O-C=O |
| C1s F | 289.78 | O-C=O (Adventitious carbon contamination) or π-π* transitions |
| N1s A | 400.05 | Pyrollic-N(-NH-) |
| N1s B | 402.21 | Pyridinic-N(=N-) |
